# Oncostatin M, an Underestimated Player in the Central Nervous System

**DOI:** 10.3389/fimmu.2019.01165

**Published:** 2019-05-29

**Authors:** Evelien Houben, Niels Hellings, Bieke Broux

**Affiliations:** Department of Immunology, Biomedical Research Institute, Hasselt University, Diepenbeek, Belgium

**Keywords:** Oncostatin M (OSM), central nervous system, cell biology, homeostasis, pathology

## Abstract

For a long time, the central nervous system (CNS) was believed to be an immune privileged organ. In the last decades, it became apparent that the immune system interacts with the CNS not only in pathological, but also in homeostatic situations. It is now clear that immune cells infiltrate the healthy CNS as part of immune surveillance and that immune cells communicate through cytokines with CNS resident cells. In pathological conditions, an enhanced infiltration of immune cells takes place to fight the pathogen. A well-known family of cytokines is the interleukin (IL)-6 cytokine family. All members are important in cell communication and cell signaling in the immune system. One of these members is oncostatin M (OSM), for which the receptor is expressed on several cells of the CNS. However, the biological function of OSM in the CNS is not studied in detail. Here, we briefly describe the general aspects related to OSM biology, including signaling and receptor binding. Thereafter, the current understanding of OSM during CNS homeostasis and pathology is summarized.

## Introduction

A well-orchestrated transmission of signals is crucial to maintain and restore homeostasis in humans. A class of messenger molecules that play an important role in interaction and communication of cells in the immune system, are cytokines. They are produced by immune cells but also by other resident cells of the human body as a response to changes in their microenvironment. The central nervous system (CNS) was long believed to be immune privileged. However, in the last decades it has become clear that communication between the CNS and the immune system is very important, even during homeostasis, and that it needs to be strictly regulated.

Many cytokines are thoroughly characterized in the CNS, yet others remain less studied, including oncostatin M (OSM). In 1991, OSM was categorized as a member of the interleukin (IL)-6 cytokine family (1, 2). Next to OSM and IL-6, this family of cytokines includes IL-11, IL-27, leukemia inhibitory factor (LIF) ciliary neurotrophic factor (CNTF), cardiotrophin-1 (CT-1), cardiotrophin-like cytokine (CLC), neuropoietin (NP) and IL-31 (3, 4). These cytokines all signal through a multi-unit receptor complex, containing the common glycoprotein 130 (gp130) subunit, except for IL-31 which binds to the OSM receptor beta (OSMRβ)/IL-31 receptor alpha (IL-31Rα) complex (5). Because of the related receptor complexes, signaling properties are shared by the members of the IL-6 family. For more insights see (4, 6–9). Here, we briefly describe the biological effects of OSM and summarize the current understanding of OSM activity in the CNS.

## OSM Biology

OSM binds to the heterodimeric OSM receptor (OSMR), consisting of the gp130/OSMRβ complex, also referred to as OSMR type II in humans. Moreover, in humans and rats, OSM signaling is also possible through the LIF receptor (LIFR), consisting of the heterodimer gp130 and LIF receptor beta (LIFRβ), also known as OSMR type I in humans (4, 10). However, in mice, murine OSM (mOSM) does not transmit signals through the LIFR (11, 12). Only extremely high concentrations of mOSM may lead to weak LIFR signaling (11). While there are reports on mOSM signaling via the LIFR in mouse osteocytes (13, 14), no literature hints to LIFR activation by mOSM in neural cells. Cross-species activities of OSM in mice, rats and humans are also possible (summarized in Table 1) (10, 15). Understanding of the cross-species activities is crucial for proper interpretation of results obtained in experimental studies. For example, experiments in which OSM only signals via the LIFR show receptor and cell signaling of LIF rather than that of OSM.

**Table 1:** Cross-species signaling of OSM via the LIFR and the OSMR in mice, rats and humans.

	**Mouse**		**Rat**		**Human**	
	**mLIFR**	**mOSMR**	**rLIFR**	**rOSMR**	hLIFR^*^	hOSMR^**^
Mouse OSM (10–12, 15)	**–**	**+**	–	+	–	–
Rat OSM (10)	–	+	**+**	**+**	+	–
Human OSM (10, 12)	+	–	+	–	**+**	**+**

* OSMR type I;

** *OSMR type II*.

OSM contains BC loops which form a steric hindrance for OSMRβ and LIFRβ. Therefore, OSM first binds with gp130 to subsequently recruit OSMRβ or LIFRβ (16). In both cases, formation of the heterodimeric complex leads to activation of different signaling cascades [extensively reviewed in (4, 6, 7, 17)]. First, receptor binding can activate Janus kinase (JAK)s, JAK1, JAK2 and Tyk2, which recruit signal transducer and activation transcription (STAT)s, STAT1, STAT3, STAT5, and STAT6. The activated STATs translocate to the nucleus to induce transcription of target genes. Second, activation of the LIFR and OSMR can also induce the mitogen activated protein kinases (MAPK) cascade. The different MAPKs involved are extracellular signal-regulated kinases 1 and 2 (ERK1/2), p38 and c-jun N-terminal kinases (JNK) (18). Finally, activation of the phosphatidylinositol-3-kinase (PI3K)/Akt pathway and the protein kinase C delta (PKCδ) have been described after OSMR activation (19, 20). These different signaling pathways lead to the diverse nature of OSM in various cell types and environmental conditions.

## OSM in the CNS

**Figure 1:**
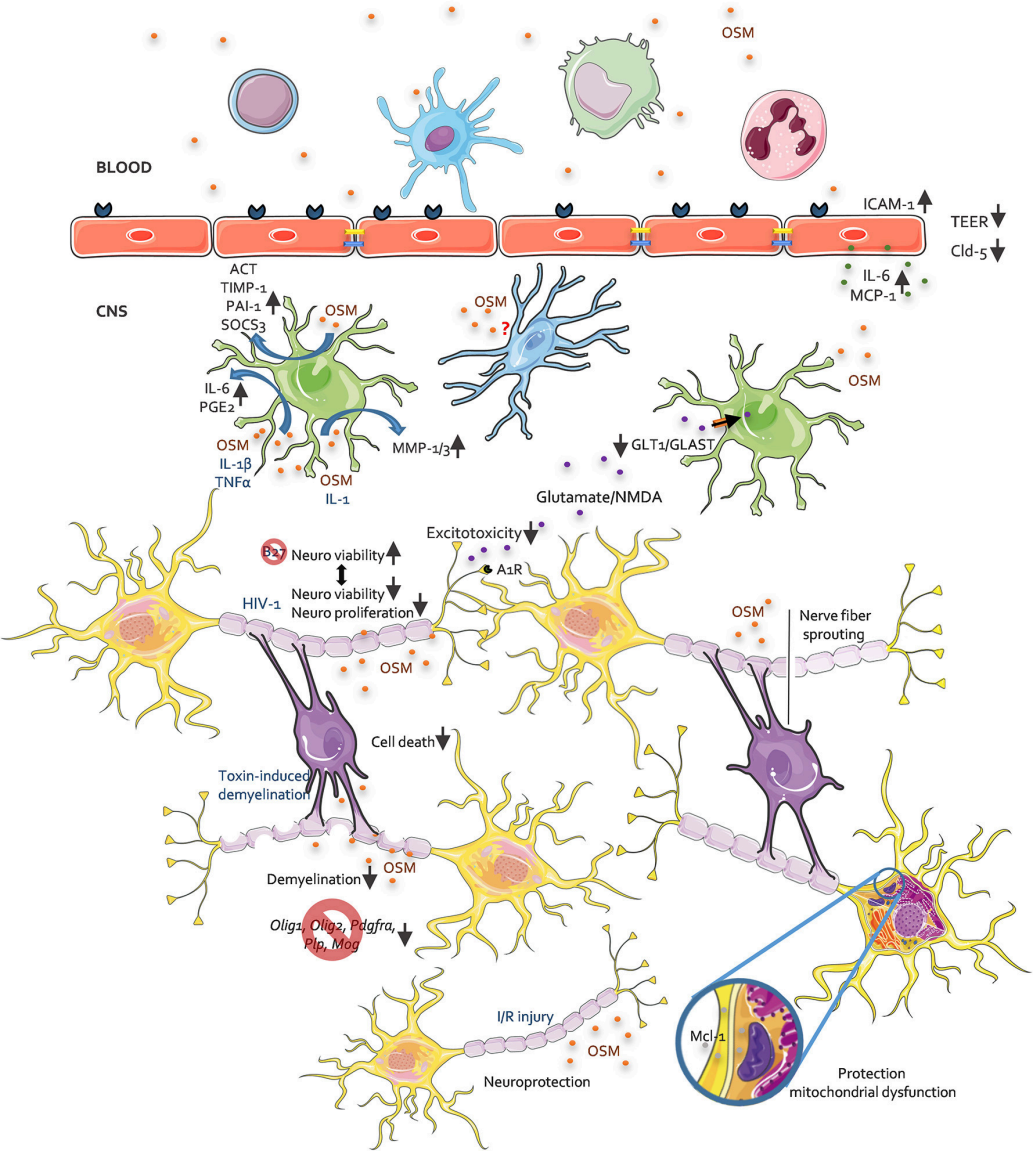
The role of OSM in CNS pathology, an overview. This figure depicts all reported activities of OSM on different CNS resident cells as investigated in *in vitro* and *in vivo* studies. For further details see accompanying text.

The role of OSM has already been specified in joint, skin, lung, and vascular homeostasis and disease [reviewed in (4, 7, 19)]. Also in cancer, depending on the cancer type, various actions of OSM are reported (4, 7, 19). Yet, less is known about the role of OSM in the CNS. Here, we summarize the reports for which OSM is described in the healthy and pathological CNS (Figure 1).

### Source of OSM and OSMRβ Expression in the CNS

Many cells of the immune system, i.e. dendritic cells, neutrophils, monocytes/macrophages, and T-cells, have been identified as a source of OSM (21–23). Hematopoietic cells of the bone marrow also produce OSM, regardless of inflammation (24). In the CNS, OSM is expressed by different cell types, namely neurons, astrocytes and microglia (25–27). In pathological conditions, such as multiple sclerosis (MS), OSM expression in the CNS is increased, in part by OSM production via infiltrating leukocytes (25, 28). With regard to expression of OSMRβ in the CNS, the first reports described expression of *OSMR*β RNA in most regions of the murine CNS (forebrain, cortex, midbrain, hindbrain and spinal cord) (29, 30). Later reports investigated the cellular specificity of OSMRβ expression, and indicated that the OSMRβ protein is expressed on neurons (31), astrocytes (31–34), endothelial cells (33), and oligodendrocytes (31). For microglia conflicting reports are found and discussed later. In addition, *OSMR*β transcripts are also present in epithelial cells of the pia mater and the choroid plexus (29, 35). The extensive expression of the OSMRβ in the brain implies important CNS related effects of OSM in different cell types both in health and disease.

### Effects of OSM on Neural Cells

In physiological conditions, OSM has been implied in the homeostasis of neural precursor cells (NPCs). NPCs are a pool of cells for the continuous production of new neural cells, located in the subventricular zone (SVZ) (36), hippocampus (37), and olfactory bulb (38) in the adult mammalian brain. In mice, the OSMR is expressed on a subpopulation of NPC in the SVZ and in the subgranular zone of the dentate gyrus within the hippocampus (39). Functionally, OSM induces *in vitro* repression of neurosphere formation, indicating inhibition of NPC proliferation isolated from the SVZ and olfactory bulb, (39) while NPCs isolated from SVZ, olfactory bulb and hippocampus of OSMRβ knock-out animals lead to enhanced formation of neurospheres (39).

In pathological conditions, the vast majority of papers report neuroprotective effects of OSM. To start, OSM inhibits N-methyl-D-aspartate (NMDA)-induced excitotoxicity in a dose-dependent way. This effect is even more pronounced after pre-treatment with OSM (40). Neuroprotective effects against excitotoxicity are among others mediated by inhibitory adenosine A_1_ receptors (A_1_Rs), suppressing excitatory transmission (41). Inhibition of glutamate-induced excitotoxicity by OSM is completely abolished after A_1_Rs blockage and knockout, indicating the requirement of adenosine A_1_R function for neuroprotection (42). Also, a protective effect of OSM is observed after amyloid beta-peptide (Aβ) induced neurotoxicity (43), known to cause mitochondrial dysfunction in Alzheimer's disease (44). Furthermore, OSM protects against 3-nitropropionic acid induced mitochondrial dysfunction in rat cortical neurons through induction of myeloid cell leukemia-1 (Mcl-1). Mcl-1 enhances mitochondrial respiration and ATP production (43) and is described as an anti-apoptotic protein with neuroprotective functions (45, 46). Since rat neuronal cells and rOSM are used in these experiments, both the involvement of LIFR and OSMR signaling needs to be considered. Moreover, we reported that OSM enhances neuronal cell viability after withdrawal of B27, a vital supplement for growth and differentiation of primary neurons and enhances neurite outgrowth *in vitro* (47). Only one study reported a potential neurotoxic effect of OSM. In this study, neuronal cell growth was inhibited when cultured in the presence of the secretome of peripheral blood mononuclear cells (PBMC) from HIV-1-infected patients. Analysis of the secretome, identified OSM as the key molecule involved in inhibition of neuronal proliferation and viability (48). Another study reported an indirect neurotoxic activity of OSM by inducing TNF-α secretion by microglia (49). Altogether, we can conclude that OSM has been widely reported to have a direct neuroprotective activity. However, indirect neurotoxic effects are possible and need to be kept under consideration.

Astrocytes usually prevent neuronal excitotoxicity via sequestration of extracellular glutamate through the glutamate aspartate transporter (GLAST/EAAT1) and glutamate transporter-1 (GLT-1/EAAT2) (50). OSM downregulates the expression of these receptors on astrocytes, leading to reduced glutamate uptake and consequently, excitotoxic injury (26). Astrocytes also secrete different molecules in response to OSM. Plasminogen activator inhibitor-1 (PAI-1) and α_1_-antichymotrypsin (ACT) (51) expression is induced by OSM in astrocytes. Co-treatment of OSM and IL-1, leads to matrix metalloproteinase (MMP)-1 and MMP-3 production by astrocytes (52). Moreover, OSM works synergistically with the pro-inflammatory cytokines, IL-1β and TNF-α, to induce IL-6 (53) and prostaglandin E_2_ (PGE_2_) (54) production in human astrocytes. These OSM-induced astrocytic molecules are linked to pro-inflammatory and tissue remodeling processes. However, OSM also induces astrocytic secretion of tissue inhibitor of matrix metalloproteinase-1 (TIMP-1) (55) and SOCS3 (32), which quench inflammation. Therefore, the net outcome of OSM signaling in astrocytes depends on the microenvironment and other cytokines present herein.

For microglia/macrophages, contradictory reports are present about OSMRβ expression and therefore the effect of OSM on these cells. Different research groups do not observe OSMRβ expression in primary mouse microglia (34, 42), nor in the C8-B4 microglia cell line (34). Moreover, no phosphorylation of STAT1 or STAT3 is observed in microglia after OSM treatment (34). In contrast, tumor necrosis factor-α (TNF-α) and nitric oxide (NO) production is reported after NF-κB pathway stimulation via OSM treatment of primary microglia and the BV2 microglia cell line (49). Yet, others did not see OSM-induced activation of the NF-κB pathway nor OSM-induced NO production in microglia (34). Moreover, our group found OSMRβ expression on Iba-1^+^ cells, a marker for both microglia and macrophages, in naive and cuprizone treated animals (31). In naive animals, no infiltration of macrophages is expected, yet perivascular, meningeal and choroid plexus macrophages are present (56). In the cuprizone challenged mice, macrophages infiltrate the brain (57). Therefore, it is possible that the Iba-1^+^ cells are macrophages. However, Hsu and colleagues did not detect OSMRβ in bone marrow-derived macrophages and the RAW 264.7 macrophage cell line (34). Yet, in other tissues, OSMRβ expression is seen on macrophages, i.e. in adipose tissue and atherosclerotic lesions (58, 59). In conclusion, more research is needed to address whether OSMR signaling is active in microglia and macrophages.

The blood brain-barrier (BBB) is very important to protect the brain from unwanted intruders. On human cerebral endothelial cells (HCECs), expression of OSMRβ, but not LIFRβ, is seen, despite low RNA levels of LIFRβ (25). The latter implies that OSM only signals through the OSMR type II in HCECs. OSM treatment increases the percentage of HCECs expressing intracellular adhesion molecule-1 (ICAM-1), yet no effect on vascular cell adhesion molecule-1 (VCAM-1) expression is detectable (25). Next to adhesion molecules, OSM augments the secretion of IL-6 and monocyte chemotactic protein-1 (MCP-1) in HCECs (25). This effect is further enhanced after co-treatment of HCECs with OSM and TNF-α (25). Also, a decreased BBB permeability is attributed to persistently high activation of the JAK/STAT3 signaling pathway (60). Here, rat brain capillary endothelial cells (RBECs) were treated with mOSM, implying OSMR and not LIFR signaling as indicated in Table 1 (10). Both increased permeability for sodium fluorescein and decreased transendothelial electrical resistance (TEER) are seen in mOSM treated RBECs (60, 61). Moreover, delocalization of the tight junction molecules, claudin-5 and zonula occludens-1 (ZO-1) is apparent after OSM treatment (60). Together, these studies imply a pro-inflammatory state of BBB-ECs when treated with OSM.

Finally, for oligodendrocytes and myelination, protective effects of LIF have been described [reviewed in (62, 63)] and tested preclinically via therapeutic delivery through nanoparticles or lentiviral vectors (64, 65). However, only a few *in vivo* studies reported on the role of OSM on oligodendrocytes and its repair mechanisms after myelin damage. These results are described in the next section. Overall, both protective and detrimental effects on cells of the CNS are described for OSM. Even though, the described *in vitro* experiments investigated the effect of OSM on distinctive cell types, the interplay between different cells is more complex and needs to be studied using *in vivo* models.

### OSM in Murine Models of CNS Pathology

Murine neurological disease models are used to investigate the biological role of OSM in a more complex *in vivo* setting and to further allow development of OSM based treatment strategies based on these insights. Inducing disease in these models can influence the level of OSM and OSMRβ expression. Indeed, upregulation of *OSM* and *OSMR*β in whole spinal cord is observed in the mouse spinal cord hemisection model (47). During cuprizone-induced demyelination increased OSMRβ expression is seen mainly on astrocytes and Iba-1^+^ cells (microglia/macrophages) (31). In contrast, middle cerebral artery occlusion (MCAO)/reperfusion reduces expression of OSMRβ on neurons in brain areas of disturbed perfusion, i.e. ipsilateral cortex and striatum (66).

To test the effect of OSM signaling, both OSM treatment and transgenic mice [OSMRβ overexpressing animals or OSMRβ knock-out (KO) mice] can be used. While OSM KO and OSMRβ KO mice are healthy and fertile, phenotypical changes observed are a disturbed hematopoiesis in both KO strains (67, 68) and severe obesity upon a high-fat diet in OSMRβ KO animals (69). It is unknown whether there is a phenotype in the CNS, since no neurological deficits are reported to date. For the OSMRβ KO mice, the IL-31 receptor consisting of OSMRβ/IL-31Rα is also affected. However, to date there are no reports of IL-31 signaling in the CNS, only the presence of the IL-31 receptor is described in dorsal root ganglia (70–73).Therefore, it is difficult to evaluate the effect of disturbed IL-31 signaling in CNS studies using OSMRβ deficient mice.

OSM was studied in the experimental autoimmune encephalomyelitis (EAE) mouse model, a MOG autoreactive T-cell mediated model for MS. Mice receiving intraperitoneal injections with OSM did not develop any sign of paralysis, the clinical outcome of EAE induction (74). The absence of symptoms was in agreement with limited immune cell infiltration in the brain of these animals (74). However, it needs to be mentioned that human OSM was used to treat the animals. Therefore, LIFR signaling and not OSMR signaling is studied here as indicated in Table 1. Indeed, systemic LIF treatment of EAE mice reduces the disease symptoms (75). We have shown that local overexpression of OSM through lentiviral vectors reduces cell-death of oligodendrocytes in the cuprizone model and therefore limits subsequent demyelination. The latter is linked to a reduced microglial response, increased IL-4 expression and M2 polarization (31). OSM-induced M2 polarization is also seen in other organ/tissue models, i.e. lung, adipose tissue (58, 76–78) and cancer models (79, 80). In line with the cuprizone model, OSM treatment in the ethidium bromide (EtBr) toxin-induced demyelination model counteracts a reduction in mRNA expression of oligodendrocyte precursor marker (*Pdgfra*), oligodendrocyte lineage transcription factors (*Olig1* and *Olig2*) and myelin genes (*Plp* and *Mog*) indicative for oligodendrocyte and myelin sparing (33). Moreover, neuroprotective effects are reported *in vivo*. Local OSM treatment in a spinal cord injury (SCI) mouse model improves functional recovery, and histological analysis reveals a reduced lesion size with less astrogliosis, less CD4^+^ T cell infiltration and an increased nerve fiber sprouting (47). Also, stereotaxic injection of NMDA together with OSM reduces the NMDA-induced lesion volume in a model of neurotoxic injury. This could be attributed to a reduced expression of the NR2C subunit of the NMDA-receptor and attenuated increase of intracellular calcium, preceding NMDA-induced cell death (40). Correspondingly, OSMRβ overexpression in neurons improves stroke outcome following ischemia/reperfusion (I/R)-induced cerebral injury (66). The latter is due to a protective role of OSMRβ against neuronal apoptosis via JAK2/STAT3 signal activation, leading to transcription of genes involved in neuronal survival (66). When mice lack OSMRβ, opposite effects on neurons and oligodendrocytes are described. OSMRβ deficiency leads to an increased infarct volume and more severe neurological deficits after I/R damage (66) and an aggravation of demyelination in the cuprizone model (31). Taken together, these studies indicate protective effects of OSM signaling in different mouse models for neurodegenerative diseases, with both oligodendrocytes and neurons being directly or indirectly protected by OSM. The most important intermediate players in these processes are astrocytes and microglia/macrophages.

## Conclusion

Within the CNS, the major cellular sources of OSM are astrocytes, neurons, microglia and infiltrating immune cells. OSM can signal though both the LIFR (OSMR type I in humans) and OSMR (OSMR type II in humans) in humans and rats, while in mice, only signaling via the OSMR is possible. Since OSM can signal via two receptors, caution should be taken when interpreting research findings, because OSM-mediated effects can be attributed to LIFR and/or OSMR signaling, depending on the model and species used. With regard to the effect of OSM on neurons, the majority of papers report protective effects of OSM. For oligodendrocytes, astrocytes, and the BBB a limited amount of studies is available. OSM has protective effects at the level of myelination, which is very important for good signal transduction and protection of axons. For astrocytes, there is a role in excitotoxicity and secretion of inflammatory molecules. Finally, at the level of the BBB a pro-inflammatory readout is observed. To conclude, OSM can exhibit different functions depending on the variety of cell types that express the receptor and the cellular and molecular microenvironment. While *in vivo* models demonstrated that OSM has beneficial effects in the diseased CNS, more research is warranted to reveal the true role of OSM in the CNS.

## Author Contributions

All authors listed have made a substantial, direct and intellectual contribution to the work, and approved it for publication.

### Conflict of Interest Statement

The authors declare that the research was conducted in the absence of any commercial or financial relationships that could be construed as a potential conflict of interest.
